# Assembly of Tomato Rhizobacteria from Different Functional Groups Improves Seedling Photosynthesis and Growth

**DOI:** 10.3390/plants12234000

**Published:** 2023-11-28

**Authors:** Yi Zhao, Yingqi Hong, Peng Wang, Yirong Gou, Rensen Zeng, Qianrong Zhang, Dongmei Chen, Yuanyuan Song

**Affiliations:** 1 Key Laboratory of Ministry of Agriculture and Rural Affairs of Biological Breeding for Fujian and Taiwan Crops, Key Laboratory of Ministry of Education for Genetics, Breeding and Multiple Utilization of Crops, College of Agriculture, Fujian Agriculture and Forestry University, Fuzhou 350002, China; zhaoyipgpr@163.com (Y.Z.); hyq985676490@163.com (Y.H.); fafuwp@163.com (P.W.); yr2654946183@126.com (Y.G.); rszeng@fafu.edu.cn (R.Z.); 2 Key Laboratory of Crop Biotechnology of Fujian Higher Education Institutes, Fujian Agriculture and Forestry University, Fuzhou 350002, China; 3 Crops Research Institute, Fujian Academy of Agricultural Sciences, Fuzhou 350002, China; zhangqianrong@faas.cn

**Keywords:** plant growth-promoting rhizobacteria, rhizosphere, tomato, synthetic rhizobacterial assembly, continuous cropping obstacle

## Abstract

The rhizosphere harbors abundant plant growth-promoting rhizobacteria (PGPR) that are vital for plant health. In this study, we screened growth-promoting bacteria from tomato rhizosphere soil, verified their functions, and constructed the optimal combination of growth-promoting bacteria for promoting tomato growth. Furthermore, the effects of these bacteria on various physiological and biochemical parameters of tomato plants were evaluated. A total of 36 strains of rhizobacteria were isolated from tomato rhizosphere soil and their abilities to produce indole-3-acetic acid (IAA), solubilize phosphate and iron carriers were assessed. The bacterial strains with the highest capacities for IAA production (R62, R317), phosphate solubilization (R41, R219), and siderophore production (R25, R325) were selected to form three bacterial combinations: R62 + R219 + R317 + R325 (T1), R62 + R325 (T5), and R317 + R325 (T8). Fifteen days after inoculation, all three combinations showed a stimulatory effect on seedling growth compared to the un-inoculated control. Inoculation with T1, T5 and T8 increased the seedling vigor index by 173.7%, 204.1%, and 168.7%, respectively. Compared to the un-inoculated control, the T1 combination increased the activities of polyphenol oxidase, peroxidase, and the net photosynthetic rate by 132.7%, 18.7%, 58.5%, and upregulated the relative expression levels of the photosynthetic assimilation-related genes *RbcL*, *RbcS*, *FBPase* and *FDA* by 22.2-, 6.6-, 1.95-, and 2.0-fold, respectively. Our findings provide a potential for constructing rhizobacterial combinations of different functional groups for improving crop growth.

## 1. Introduction

Tomatoes, renowned for their nutritional value and diverse culinary applications, hold an integral place in agriculture worldwide [1]. However, sustainable cultivation remains a significant challenge, particularly due to the practice of monoculture—the continual growth of tomatoes in the same plot year after year [2]. While these practices simplify farming operations and standardize yields, they pose considerable challenges, including soil degradation, nutrient exhaustion, and a heightened vulnerability to diseases and pests. The impact of monoculture on soil health is profound [3,4,5]. Over time, the continuous cropping of tomatoes depletes vital soil nutrients, leading to a gradual decrease in soil fertility [6]. As tomatoes require a range of nutrients for optimal growth, this nutrient depletion can significantly reduce crop yields, threaten food security, and in extreme cases, render the land unfit for further cultivation [7].

Amid these challenges, potential solutions emerge from the intricate biological world surrounding plant roots: the rhizosphere [8]. The rhizosphere is a dynamic zone teeming with microbial life, wherein complex interactions between plants and microorganisms take place. Among these microorganisms, plant growth-promoting rhizobacteria (PGPR) hold significant promise as a potential solution to the continuous cropping obstacle presented by continual tomato monoculture [9].

PGPR are a diverse group of bacteria that colonize plant roots and rhizosphere soils, and promote plant growth by enhancing nutrient acquisition, producing plant growth hormones, and protecting plants from diseases. These bacteria can be found naturally in the soil, but their populations can be boosted through the use of biofertilizers, which aresubstances containing living microorganisms that enhance the host plant’s nutrient supply when applied to seeds, plant surfaces, or soil [10]. One way that PGPR enhances nutrient acquisition is by transforming nutrients into a form that plants can easily absorb. For example, they can solubilize phosphorus, a key nutrient that is often present in soils but not always readily available to plants [11]. Additionally, some PGPR can facilitate nitrogen fixation, a process that converts atmospheric nitrogen into a form that plants can use for growth [12]. These activities not only improve plant health and productivity, but also contribute to the sustainable management of soil fertility. Beyond nutrient acquisition, some strains of PGPR produce plant growth hormones such as auxins, gibberellins, and cytokinins, which directly stimulate plant growth. These hormones can promote cell division, root formation, and bud growth, leading to overall enhanced plant growth [13]. This aspect of PGPR activity is particularly beneficial in early plant development stages and can lead to improved crop establishment and growth.

Furthermore, PGPR can serve as a powerful ally against soil-borne diseases. Some beneficial bacteria can outcompete pathogenic bacteria for resources, thereby reducing their populations in the rhizosphere [14]. Others produce antimicrobial substances that directly inhibit pathogen growth [15]. Yet another group of PGPR can induce systemic resistance in plants, essentially ‘priming’ the plant’s immune system so that it can respond more robustly to pathogen attacks [16]. This multi-pronged approach not only helps control disease, but also reduces reliance on chemical pesticides, thus contributing to a more sustainable and eco-friendly agricultural system. Despite these benefits, the application of PGPR is not without its complexities. The introduction of non-native bacteria can sometimes lead to unintended ecological consequences, such as the disruption of local microbial communities [17]. Moreover, the effectiveness of PGPR can vary depending on numerous factors, including the soil type, environmental conditions, and the presence of other microbes in the soil. It is also worth noting that while some PGPR strains have beneficial effects on plant growth and health, others can be pathogenic or have neutral effects, underscoring the need for careful strain selection and management [18]. Thus, while the potential use of PGPR in overcoming the challenges of tomato monoculture is clear, their application must be guided by thorough research and understanding. Future studies should aim to elucidate the complex interactions between PGPR, plants, and their environment, with a focus on identifying the most effective strains and application methods in different agricultural contexts [19].

Although the challenge of reducing the use of chemical fertilizers without compromising tomato yields is enormous, rhizosphere bacteria offer a promising avenue for mitigation. With further research and the judicious application of PGPR, we can improve the sustainability and resilience of tomato cultivation [20]. The aim of this study was to screen beneficial rhizobacteria with the ability to promote growth and photosynthesis in tomato seedlings. Six rhizobacterial strains with different functions were selected to form three bacterial combinations for the growth promotion of tomato. Our findings offer a fresh perspective on the advancement of biofertilizers.

## 2. Results

### 2.1. The Selected Strains Show Traits Beneficial for Plant Growth

**Figure 1:**
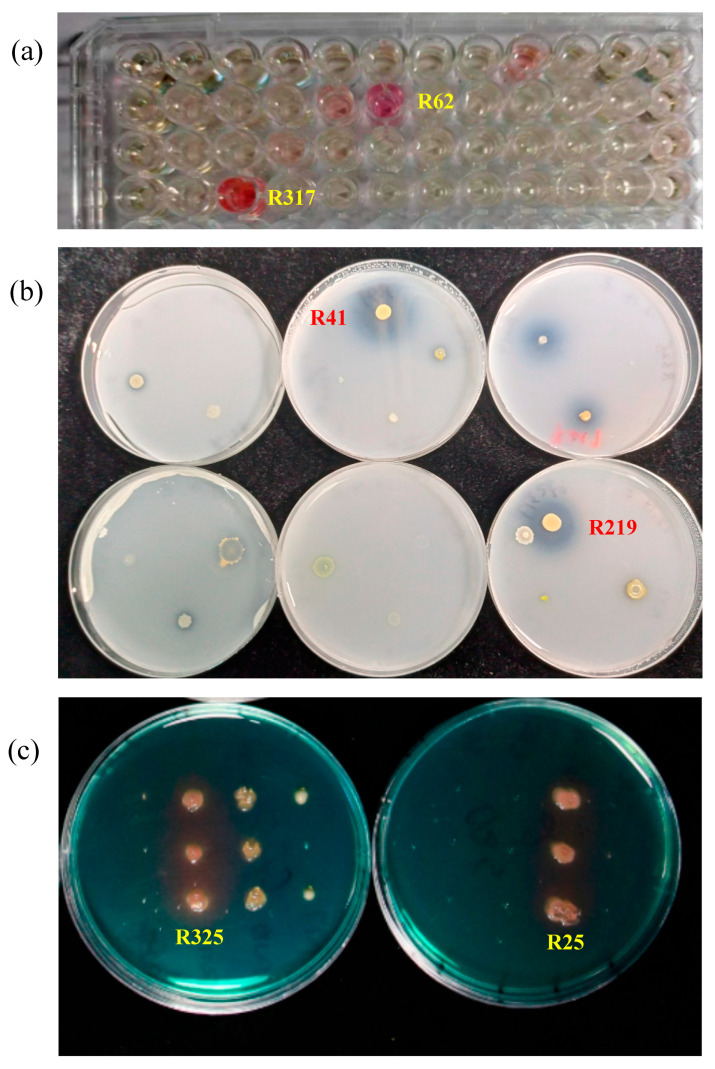
IAA-producing, phosphorus-solubilizing, and iron-carrier-producing capacities of the 36 PGPRs. (**a**) Schematic diagram of Salkowski colorimetric reaction (the intensity of a chromogenic reaction indicates the strain’s ability to generate IAA); (**b**) Capacity of some isolates to solubilize phosphate (a transparent circle observed around the phosphate-solubilizing bacteria and the size of the transparent circle indicate the capacity for phosphate solubilization); (**c**) Capacity of isolates to produce siderophores (the size of the pale yellow circle represents the capacity for siderophore production).

A total of 36 different strains of bacteria were isolated from the rhizosphere soil of tomato. Proteobacteria accounted for 15 species, followed by Actinobacteria (10 species), Firmicutes (8 species) and Bacteroidetes (3 species) (Table 1). After a 30 min reaction in the absence of light, the color of the bacterial fermented supernatant and Salkowski reagent mixture was determined. The intensity of color change into pink represented the bacterial strain’s ability to produce IAA. The most obvious color reaction was observed in strains of R62 and R317 (Figure 1a), and their IAA production was 7.51 µg·mL^−1^ and 9.92 µg·mL^−1^ in 24 h (Table 2), respectively. The selected strains were inoculated on Pikovskaya’s agar and cultured for 7 days to observe the strains and their transparent circles (Figure 1b). The phosphorus solubilizing energy was determined by comparing the ratio of the diameter of the phosphorus solubilization circle (D) to the diameter of the colony (d). Strains of R219 and R41 were the most potent strains, and the ratios of the diameter of the phosphorus solubilization circle (D) to the diameter of the colony (d) were 3.33 and 3.00 (Table 3), respectively. A total of 24 strains produced orange siderophore halos via plate initial screening (Figure 1c). R325 and R25 had the highest OD_680_ values of 3.1275 and 3.0795, respectively, suggesting that the two bacteria displayed the strongest ability to produce siderophores (Table 4).

### 2.2. Effect of Bacterial Combinations on the Growth of Tomato Seedlings

Based on the results showing that R317 and R62 had the strongest IAA production ability, R219 and R41 had the strongest phosphorus solubilizing ability, and R325 and R25 showed the strongest siderophore production ability, the six strains were used for the construction of combinatorial growth promoters. First, seven combinations of growth-promoting bacteria were set, and MIX represents the combination of six strains (R317, R62, R219, R41, R325 and R25): MIX-R25 (indicating exclusion of R25), MIX-R41 (indicating exclusion of R41), MIX-R62 (indicating exclusion of R62), MIX-R219 (indicating exclusion of R219), MIX-R317 (indicating exclusion of R317), and MIX-R325 (indicating exclusion of R325); that is, the strains after indicate the combined bacteria without the addition of one of the strains. In future experiments, combinations that do not promote growth efficiently will be eliminated as they may not enable the bacteria to produce synergistic effects.

After two rounds of inoculation tests (Tables S2 and S3), R25 and R41 with a poor growth promotion effect were eliminated, and two combinations with the highest seedling index (R62 + R219 + R317 + R325 (T1) and R62 + R219 + R317 (T2)) were obtained. Six combinations of R62 + R219 (T3), R62 + R317 (T4), R62 + R325 (T5), R219 + R317 (T6), R219 + R325 (T7) and R317 + R325 (T8) were further refined.

**Figure 2:**
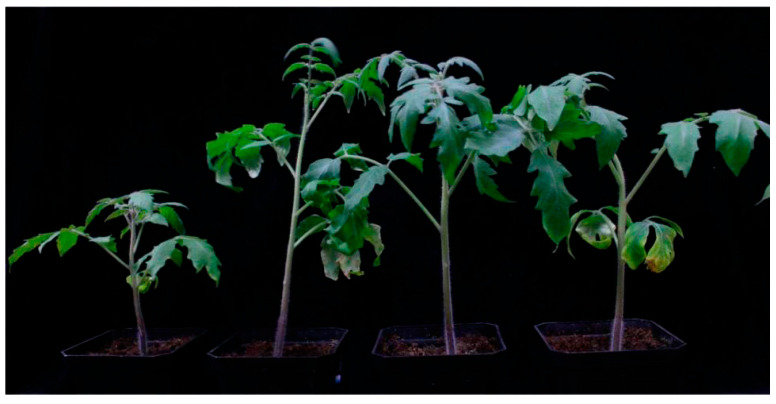
The 30-day effect of the combination bacteria T1, T5 and T8 on tomato seedlings (from left to right: CK, T1, T5, T8).

After 15 days of inoculation, the chlorophyll content of each treatment group was significantly higher than that of CK. The plant height in all PGPR-inoculated groups was significantly higher compared with the control without bacterial inoculation, except T3 (Table S4). T1, T2, T3, T4, T5, T6, T7 and T8 increased the plant height by 25.6%, 24.9%, 2.6%, 30.6%, 21.0%, 22.3%, 10.0% and 15.9%, respectively. Their inoculation increased the stem diameters by 38.1%, 32.3%, 33.0%, 42.0%, 35.2%, 35.2%, 35.5% and 30.9%, respectively. The inoculation also significantly increased the fresh weight and dry weight of the tomato seedlings. The eight combinations increased the dry weight by 137.0%, 111.9%, 99.2%, 135.4%, 152.2%, 117.3%, 103.4% and 135.5%, respectively. Based on the above results, the final combinations of growth-promoting bacteria selected were T1, T5, and T8. Compared with the un-inoculated control, T1, T5, and T8 increased the seedling index by 173.7%, 204.1% and 168.7%, respectively. The three bacterial combinations were used for subsequent trials (Table S4, Figure 2).

### 2.3. Effect of Bacterial Inoculation on Photosynthetic Parameters

**Figure 3:**
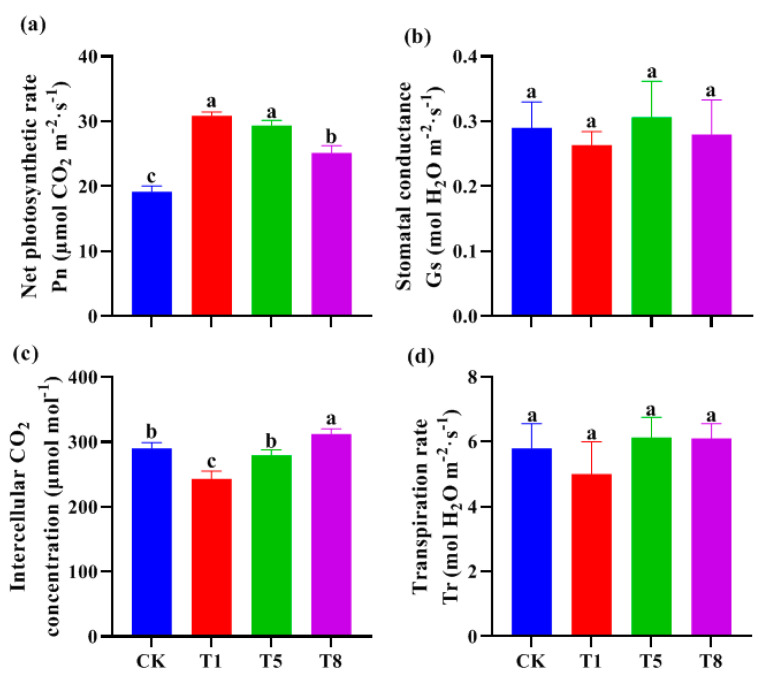
Effects of different combinations of growth−promoting bacteria on photosynthetic parameters of tomato seedlings. Three growth-promoting bacteria combinations of T1, T5 and T8 were used as treatment groups, and ddH_2_O treatment was used as the control group (CK). Each treatment had 12 tomato seedlings. After 30 days of treatment, the net photosynthetic rate (**a**), stomatal conductance (**b**), intercellular CO_2_ concentration (**c**) and transpiration rate (**d**) were measured. Data are mean ± standard error (*n* = 12). Significant differences (*p* < 0.05 using ANOVA followed by a Tukey test) among treatments are indicated by different letters above the bars.

The net photosynthetic rate (Figure 3a), stomatal conductance (Figure 3b), intercellular carbon dioxide concentration (Figure 3c) and transpiration rate (Figure 3d) of tomato leaves were measured to investigate the effects of inoculation with microbial combinations on the photosynthetic characteristics and light energy utilization rate of tomato seedlings. Compared with the un-inoculated control, the three bacterial combinations significantly increased the net photosynthetic rate of tomato seedlings. T1, T5, and T8 increased the net photosynthetic rate by 58.5%, 54.1% and 26.3%, respectively. T8 increased the intercellular carbon dioxide concentration by 8.0% (Figure 3c). There were no significant differences in the stomatal conductance and transpiration rate between the inoculated and un-inoculated control. This suggests that the combined bacteria promote tomato seedling growth by enhancing photosynthesis. 

### 2.4. Effect of Bacterial Inoculation on the Expression of Genes Involved in Photosynthetic Carbon Assimilation

**Figure 4:**
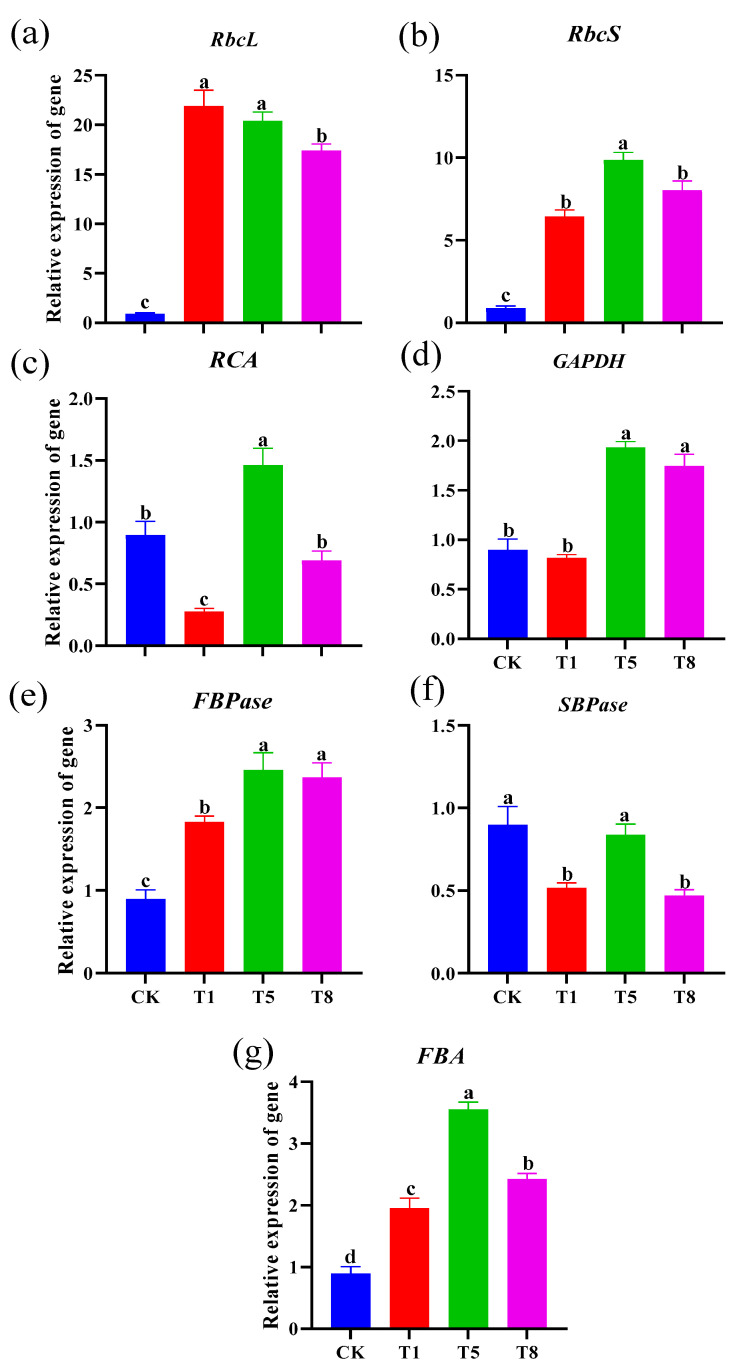
Effects of different growth-promoting bacteria combinations on the expression levels of carbon-assimilation-related genes in tomato seedlings. Three growth-promoting bacteria combinations, T1, T5 and T8, were used as treatment groups, and ddH_2_O treatment was used as the control group (CK). Each treatment had 15 tomato seedlings. The bacteria were inoculated on the roots two to three times per week. After 30 days of inoculation, the relative gene expression levels of *RbcL* (**a**), *RbcS* (**b**), *RCA* (**c**), *GAPDH* (**d**), *FBPase* (**e**), *SBPase* (**f**) and *FBA* (**g**) in different treatments were determined. Data are mean ± standard error (*n* = 15). Significant differences (*p* < 0.05 using ANOVA followed by a Tukey test) among treatments are indicated by different letters above the bars.

In order to examine the effect of inoculation with combinatorial bacteria on the photosynthesis of tomato seedlings, RT-qPCR analyses were performed on the genes (*RbcL*, *RbcS*, *RCA*) encoding the large and small subunits of Rubisco, the key enzyme for catalyzing the CO_2_ fixation process of photosynthesis (Figure 4a–c), the gene (*GAPDH*) encoding the key enzyme for the glycolytic process (Figure 4d), the gene (*FBPase*) related to the key enzyme of the dark reaction of photosynthesis (Figure 4e), an important enzyme in the circulation of the carbon source of the Calvin cycle (*SBPase*) (Figure 4f), the related genes of fructose-1,6-phosphate aldolase (*FBA*) (Figure 4g), and genes related to fructose-1,6-bisphosphate aldolase. Compared with the control group, the relative expression levels of the photosynthesis-associated genes *RbcL*, *RbcS*, *FBPase* and *FBA* were significantly upregulated by 1794.9%, 925.4%, 160.6% and 297.9% in the T5 group, respectively. T8 inoculation upregulated the expression levels of *RbcL*, *RbcS*, *FBPase* and *FBA* by 2034.8%, 701.7%, 141.1% and 141.8%, and T1 upregulated their expression levels by 2118.9%, 564.1%, 94.8% and 103.8%, respectively. T5 and T8 significantly increased the expression of *GAPDH* by 101.7% and 83.3%, respectively. T5 significantly increased the expression of *RCA* by 54.6%. These results suggest that combinatorial bacteria increase the intensity of photosynthesis by increasing the expression of genes related to Rubisco enzymes, glycolysis, dark response and the C3 pathway. 

### 2.5. Effect of Bacterial Inoculation on Defense Related Enzymes

**Figure 5:**
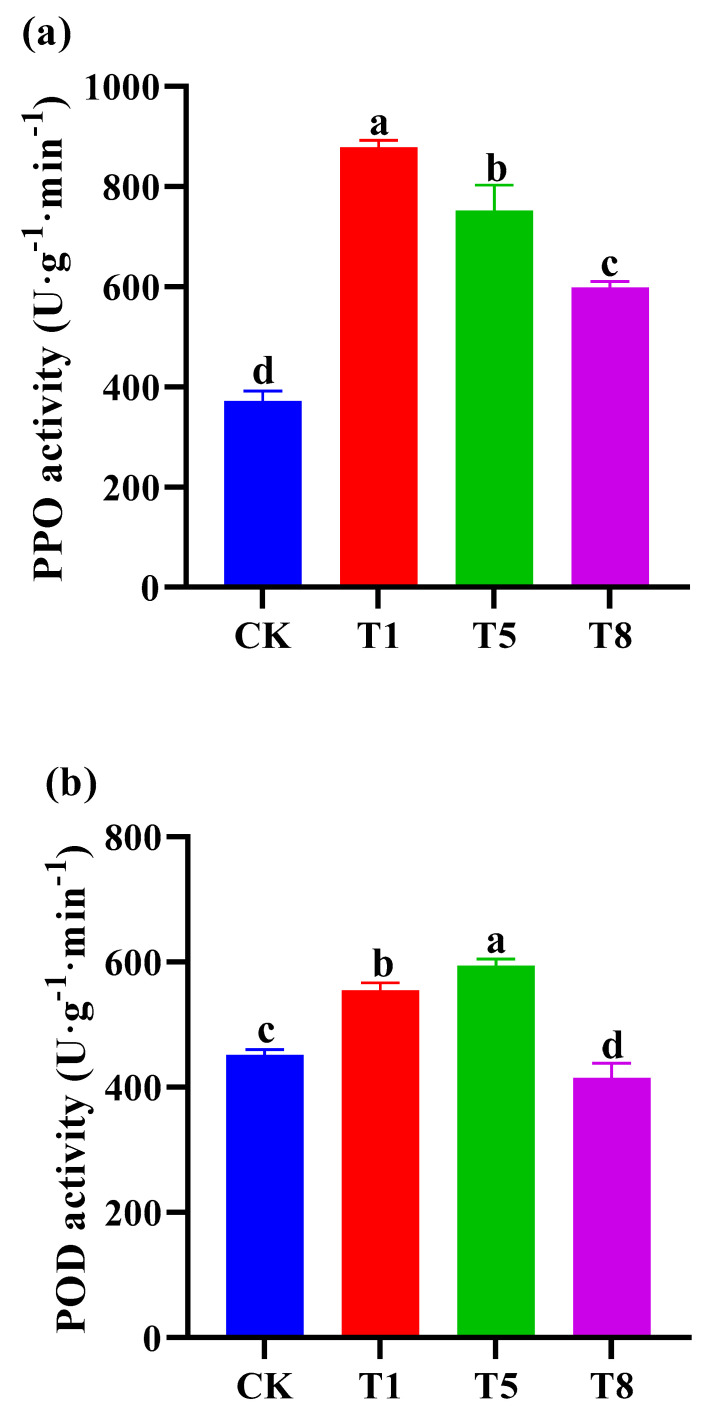
Effects of different growth−promoting bacteria combination treatments on the antioxidant and defense enzyme activities of tomato seedlings. Three growth-promoting bacteria combinations, T1, T5 and T8, were used as treatment groups, and ddH_2_O treatment was used as the control group (CK). Each treatment had 15 tomato seedlings. The bacteria were inoculated on the roots two to three times per week. After 30 days of treatment, the activities of polyphenol oxidase (**a**) and peroxidase (**b**) in different treatments were determined. Data are mean ± standard error (*n* = 15). Significant differences (*p* < 0.05 using ANOVA followed by a Tukey test) among treatments are indicated by different letters above the bars.

Peroxidase (POD) plays a protective role in both respiration and photosynthesis in plants, scavenging reactive oxygen species from the body. Polyphenol oxidase (PPO) can rapidly activate and catalyze the oxidation reaction between phenol and O_2_ to form quinone, leading to the browning of tissues and improving plant resistance. Compared with the control, all three bacterial combinations significantly increased the activity of polyphenol oxidase (Figure 5a) in tomato leaves, with T1 being the most significant by 132.7%. The POD activity (Figure 5b) in the leaves was increased by T5 and T1 by 31.3% and 18.7%, respectively. These results suggest that combinatorial bacteria can improve tomato resistance to oxidative stresses.

### 2.6. Effect of Bacterial Inoculation on Seed Germination of Tomato

**Figure 6:**
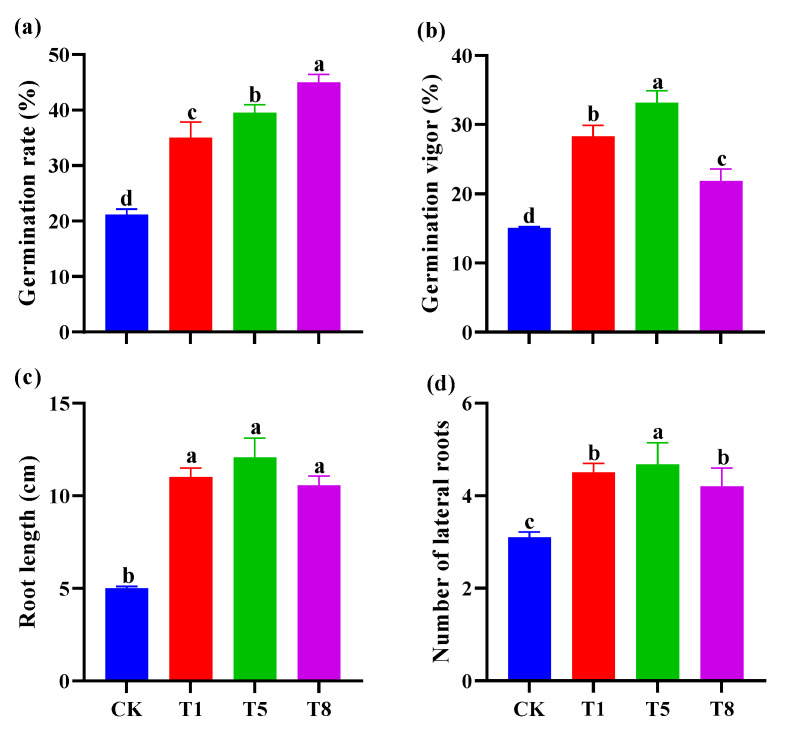

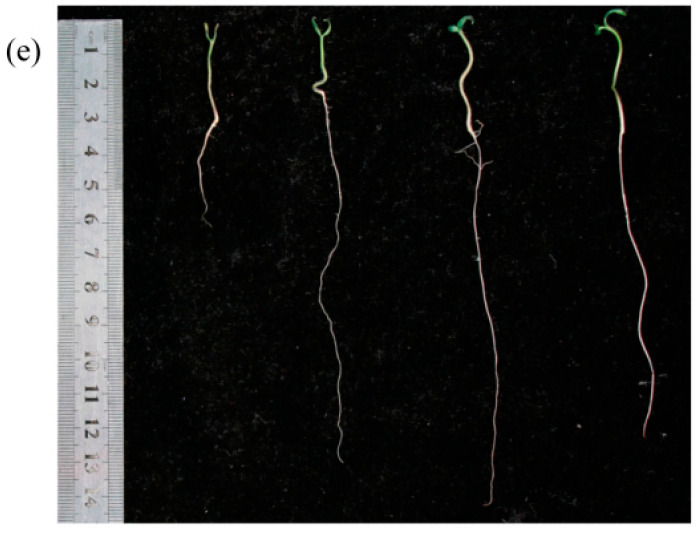
Effects of inoculation with different mixed growth-promoting bacteria on tomato seed germination and seedling growth. Three growth-promoting bacteria combinations, T1, T5 and T8, were used as treatment groups, and ddH_2_O treatment was used as the control group (CK). Each treatment had 50 tomato seedlings. The germination rate (**a**) was counted every day, and the germination vigor (**b**) was calculated at 7 days. Ten days after germination, the length of the root (**c**) and numbers of lateral root (**d**) were measured. Values are means ± standard error (*n* = 10). Different letters above bars indicate significant differences among treatments (one-way ANOVA and Tukey’s multiple range test, *p* < 0.05). (**e**) Comparison of control group and treatment group chart of seed germination at 10 days (from left to right: CK, T1, T5, T8).

PGPR not only promotes the growth and development of plants, but also improves seed germination. After seed soaking with growth-promoting bacterial combinations (T1, T5 and T8), the germination rate (Figure 6a), germination vigor (Figure 6b), as well as seedling root length (Figure 6c) and lateral root number (Figure 6d) were significantly improved. T5 showed the most significant effect on the germination potential and lateral root number, which were increased by 116.4% and 50.0%, respectively. T8 showed the most significant effect on the germination rate, which was increased by 99.9% compared with the control. There was no significant difference in root length among the three treatments.

## 3. Material and Methods

### 3.1. Bacterial Strains Used for the Studies

The donor soil was collected from the rhizosphere soil of tomato cultivation for many years in the base of Fujian Agricultural Academy of Engineering Research Institute (26°13′ N, 119°33′ E), located in Puweir Village, Xadian Town, Jin’an District, Fuzhou City (China). The rhizospheric soil was used for bacterial isolation using the serial dilution method [21]. The screening medium utilized was LB medium. Purified bacterial strains were then used for verifying functions and promoting plant growth.

### 3.2. Identification of Bacterial Strains

Bacterial strains were identified via 16S-rRNA gene sequencing. PCR amplification was performed using the universal primers F:5′-AGAGTTTGATCCTGGCTCAG-3 R:5′-GGTTACCTTGTTACGACTT-3′ [22]. The PCR conditions used for amplification were initial denaturation at 95 °C for 5 min and 35 cycles of denaturation at 95 °C for 1 min, annealing at 53 °C for 1 min and extension at 72 °C for 1 min, and final extension at 72 °C for 10 min. PCR products were detected using agarose gel electrophoresis, and single-band colonies were selected and sent to Fuzhou Shangya Biological for sequencing. After sequencing, the results were put back into the EzBioCloud platform (https://www.ezbiocloud.net/, accessed on 1 November 2020).

### 3.3. Analysis of Plant-Growth Promotion Traits

Phosphate solubilization was measured via the spot inoculation of bacteria on Pikovskaya’s agar plates supplemented with insoluble tricalcium phosphate. The formation of clear zones around bacterial colonies indicated the hydrolysis of inorganic phosphate into a plant-available form of phosphorus [23]. 

The production of indole acetic acid (IAA) was estimated using the colorimetric method [24]. Freshly grown cultures (50 µL) with OD_600_ = 0.7 were inoculated into LB broth medium (50 mL) supplemented with 0.1 g·L^−1^ tryptophan and incubated at 32 ± 2 °C at 200 rpm for 6 d. The cultures were centrifuged, and the supernatant was mixed with Salkowski reagent (2 mL of 0.5 M FeCl_3_ and 98 mL of 35% HClO_4_). The development of the pink color indicated the production of IAA. The IAA concentration was quantified by recording the absorbance at 535 nm using a spectrophotometer (Bio Tek, Burlington, VT, USA). The standard curve was plotted by using different concentrations (20, 40, 60, 80, 100, 120 µg·mL^−1^) of IAA. The experiment was carried out in triplicates.

Siderophore production was measured via the spot inoculation of bacteria on CAS (Carbon–Arginine–Succinate) agar plates. The bacteria with different colony forms in good growth states were selected and inoculated on CAS detection medium, respectively, and cultured in an incubator at 28 °C for 24 h. Orange halos appeared on the solid medium when the bacteria produced a siderophore. The preparation of the CAS plate was a follows [25]: (1) The CAS staining solution contained 1 mmol·L^−1^ chromium azurol (CAS), 4 mmol·L^−1^ hexadecyltrimethylammonium bromide (HDTMA) and 0.1 mmol·L^−1^ FeCl_3_, which were fully mixed to obtain a CAS staining solution. (2) Then, 2.427 g of Na_2_HPO_4_·12H_2_O, 0.5905 g of NaH_2_PO_4_·2H_2_O, 0.075 g of KH_2_PO_4_, 0.25 g of NH_4_Cl and 0.125 g of NaCl were weighed and thoroughly mixed to prepare 100 mL of phosphate buffer (pH 6.8). (3) Each 100 mL CAS assay medium contained 1 mL of 20% sucrose solution, 3 mL of 10% acid hydrolyzed casein, 100 μL of 1 mmol·L^−1^ CaCl_2_, 2 mL of 1 mmol·L^−1^ MgSO_4_ and 1.8 g of AGAR. Solutions (1), (2), and (3) were sterilized, cooled to 50 °C, and 5 mL of solutions (1) and (2) were added slowly to (3), respectively, to obtain a blue CAS assay medium. The equal volume of bacterial culture supernatant was mixed with CAS detection solution and reacted in the dark for 1 h. The absorbance of CAS was detected at a 680 nm wavelength. Meanwhile, the absorbance of the blank medium reacting with an equal volume of the CAS detection solution was measured [26].

### 3.4. Tomato and Soil Materials Used in the Experiment

The material used in this study was tomato (Solanum lycopersicum, Castlemart; TGRC accession number: LA2400), and the seeds were obtained from Chuanyou Li, Institute of Genetics and Developmental Biology, Chinese Academy of Sciences. Tomatoes were grown as follows: seeds were soaked in 10% hydrogen peroxide solution for 10 to 15 min. The substrate soil was sterilized at 121 °C for 30 min and then cooled to room temperature. This was then placed in a seedling box. The seeds were placed in the soil at a depth of 5 mm using forceps, sprayed with sterile water until the soil was evenly moist, placed on a light stand and turned on when white. The light conditions were 14 h/10 h, light/dark, and the room temperature was 26 °C. After 15 days, the plants were transplanted into 7 cm × 7 cm × 7 cm cubic pots. The substrate soil used for seedlings was peat soil imported from Denmark.

### 3.5. Pot Inoculation and Analysis of Growth Indicators

Bacterial inoculation started 5 days after the transplanting of tomato seedlings, which were treated every 2~3 days for a total of 15~20 days with 12 tomato seedlings per treatment. The strains were incubated with LB medium at 37 °C and 180 r·min^−1^ for 24 h on a shaker, centrifuged at 8000 r·min^−1^ for 5 min, and resuspended in sterile water to achieve an OD_600_ value of 0.8 for each bacterial suspension. In order to better explore the growth-promoting effects of each individual bacterium, equal volumes of each bacterial solution were mixed so that the total volume was 6 mL. The bacteria solution was sucked with a precision pipet tip and gently and evenly deposited around the tomato roots. An equal volume of sterile water was used as the control group. The combination with six bacteria was used as a standard to evaluate its growth-promoting effect and then each combination subtracted one bacterium for comparison with the combination of six bacteria. The rest of the combinations were evaluated in the same manner to find the best bacterial combination for plant growth promotion. 

The leaf chlorophyll contents were measured using Soil and Plant Analyzer Development (SPAD). The absorbance (SPAD value) of leaves at the wavelengths of 400–500 nm and 600–700 nm was measured using SPAD-502Plus, and the average value was calculated after measuring one value at the middle, tip and basal tip of leaves, respectively. Measurement of plant height: the length from the root to stem growth point was measured using a ruler; stem diameter: a Vernier caliper was used to measure the diameter of the lower 2/3 of cotyledons; determination of dry mass: the above-ground stems and leaves and underground roots of the tomato plants were put in an oven at 105 °C for 15 min, then dried at 75 °C to a constant mass, and then the dry mass was accurately weighed. The seedling index was calculated as follows: seedling index = (stem diameter/plant height + underground dry mass/aboveground dry mass) × whole plant dry mass.

### 3.6. Determination of Photosynthetic Parameters 

The tomato seedlings with different inoculations were cultured in the greenhouse. The second fully expanded and well-grown leaf from the top to the bottom was selected for the determination of the photosynthetic rate (Pn), stomatal conductance (Gs), intercellular carbon dioxide concentration (Ci), and transpiration rate (Tr) using a portable photosynthetic-fluorescence system (LI-6400 XT). The measurements were conducted between 9:00 am and 12:00 am in sufficient light. After the instrument parameters were stabilized, measurements were started. Each treatment had 12 seedlings and each measurement was repeated 5 times.

### 3.7. Detection of Expression Levels of Genes Involved in Photosynthetic Carbon Assimilation

After 15 days of inoculation, tomato leaves from the treatment and control groups were sampled. The leaves were ground to powder in liquid nitrogen, then total RNA was extracted using TRIzol reagent (TaKaRa, Kyoto, Japan). The first-strand cDNA was synthesized from 1000 ng of total RNA using the GoScript™ Reverse Transcription Mix, Oligo (dT), according to the manufacturer’s instructions. The reverse transcriptional reaction conditions were as follows: 25 °C, 5 min; 42 °C, 60 min; 75 °C, 15 min; and 4 °C, ∞. Quantitative reverse transcription PCR (RT-qPCR) experiments were performed to analyze the expression levels of genes encoding *RbcL*, *RbcS*, *RCA*, *GAPDH*, *FBPase*, *SBPase* and *FDA* (Table S1). The RT-qPCR reactions were carried out with 12.5 µL of the SYBR green master mix, 1 µL of cDNA, 0.2 μL (10 μM) of each specific primer, and 11 µL of RNase-free water. RT-qPCR was performed using the Step One Plus PCR instrument (Applied Biosystems). The thermal cycle reaction conditions were as follows: 95 °C, 1 min; 95 °C, 20 s; 58–60 °C, 15 s; 72 °C, 30 s; 40 cycles; and 82 °C, 1 s. The housekeeping gene actin was used as an endogenous control in the RT-qPCR experiment. The internal reference gene used was *EF*-*1a* (Table S1).

### 3.8. Assay of Activities of PPO and POD

Plants were cultured in the sterilized soils with many years of continuous tomato cropping and then inoculated with three bacterial combinations and the water control check (CK). Thirty days after inoculation, the plants were harvested to evaluate the peroxidase and polyphenol peroxidase activities. For analysis, a pair of leaves was selected from each plant and at least three plants were used for each replicate. The enzymes were extracted from 0.1 g of leaf tissues via grinding at 4 °C with 1 mL of extraction buffer, and 0.1 M sodium phosphate buffer of neutral pH for peroxidase (POD) and polyphenol peroxidase (PPO). The extracts were centrifuged at 13,000 rpm for 20 min at 4 °C and the supernatants were used as crude enzyme sources. The POD activity assays were carried out as described in the Worthington Enzyme Manual [27]. For POD activity, 2.95 mL of substrate solution (1% *v*/*v* guaiacol in 0.05 M sodium phosphate buffer of pH, 6.5 containing 100 mM H_2_O_2_) was added to 0.05 mL of crude enzyme extract, and a change in absorption was recorded at 470 nm for 4–5 min. Peroxidase activity was defined as the units of an enzyme that increase the absorbance at a 470 nm wavelength per gram of fresh weight of leaf tissue in one min (U·g^−1^ FW). For PPO activity, 2.9 mL of a 0.05 mM solution of L-tyrosine in 0.5 M sodium phosphate buffer at pH 6.5 was added to a 100 µL crude enzyme extract and the change in absorption was recorded at 280 nm for 10 min. PPO activity was defined as the units of an enzyme that produces 1 mM of quinine per min per gram of fresh weight (U·g^−1^ FW) [28].

### 3.9. Stimulatory Effect on Seed Germination of Tomato

The tomato seeds with full particles and uniform size were washed with sterile water, sterilized with 10% hydrogen peroxide solution for 1 min, and then sterilized with 75% alcohol for 60 s. The seeds were washed with sterile water 5 times and then soaked in the suspension of combined bacteria for 3 h. Seeds soaked in the same amount of sterile water for 3 h were used as controls. Fifty seeds were transferred to a dish with moist sterile filter paper and incubated at room temperature. Each treatment was performed in triplicate. Germination was counted at regular intervals every day. 

A radicle penetration of 2 mm through the seed coat was considered the initiation of germination. The number of sprouts was counted daily, and the germination potential was calculated for 10 days. After 10 days of incubation, the germination rate and germination vigor were calculated. Germination rate = S1/St × 100%; germination vigor = S2/St × 100%; S1: number of germinated seeds in 10 days, S2: number of germinated seeds in 7 days, St: total number of seeds in 7 days of experimental treatment, with 10 tomato seedlings selected from each treatment (3 replicates). The length of tomato seedlings from the bottom of the radicle to the top of the hypocotyl was measured with a ruler.

### 3.10. Data Analysis

GraphPad Prism 8.0.2 software and Microsoft Excel 2013 were used to process and plot the data. SPSS 19.0 (SPSS, Chicago, IL, USA) software was used for statistical analysis [29]. The bioassays, physiological and biochemical experiments were performed according to a completely randomized design. A one-way ANOVA and Tukey’s multiple range test (*p* < 0.05) were used to evaluate the significance of differences among treatments. The independent sample T-test was used to evaluate the significance of differences between the bacterium-inoculated group and the un-inoculated group. The normality (*p* > 0.05) of all data and the homogeneity of variance (*p* > 0.05) were confirmed through the Shapiro–Wilk test and Levene’s test, respectively, using SPSS 19.0 software.

## 4. Discussion

With the increasing prominence of environmental issues, reducing the use of chemical synthetic fertilizers and pesticides has become a consensus among people [30]. Rhizosphere engineering with plant growth-promoting microorganisms can act as a desired approach to enhancing plant productivity, promoting litter decomposition and soil fertility, and accelerating nutrient cycling [31]. In many plants such as potato, tomato, Arabidopsis, and maize, it has been shown that microbial combinations have a greater potential to promote plant growth compared to the application of single bacterial strains [32,33,34,35,36,37].

### 4.1. The Bacterial Combinations Have a More Pronounced Growth-Promoting Effect on Tomato Seedlings Compared to a Single Bacterium

The screening of rhizosphere growth-promoting bacteria primarily relies on specific microbial functions, such as phosphorus solubilization, nitrogen fixation, siderophore production, plant hormone production, and ACC deaminase activity. Typically, the most functionally potent bacterial strains are first tested in greenhouse conditions and then further evaluated in field trials [38]. Bacterial IAA producers (BIPs) can have a positive effect on root system elongation and development, thereby helping water and nutrient uptake [39]. In this study, 36 bacteria were isolated on LB medium, and R62 and R317 had the strongest IAA-producing ability (Table 2). It was found that *Pseudomonas koreensis* could produce IAA up to 65.9 (+0.03) μg·mL^−1^ and 111.6 (+0.02) μg·mL^−1^ with the addition of 0.5 mg of tryptophan [40]. While the ability of the bacteria to produce IAA in our study was lower than that of previous studies, combining growth-promoting bacteria with different functions provides a better growth-promoting effect than bacteria with only one function. Phosphate-solubilizing microorganisms (PSMs), including bacteria, have an important role in plant growth, making the unavailable insoluble sources of P available to the actively growing plants [41]. The most effective phosphorus-solubilizing bacteria obtained in this study were R41 and R219 (Figure 1b). The ability to produce siderophores has been considered an important mechanism of plant growth-promoting rhizobacteria. They are organic compounds with low molecular masses, produced by microorganisms in order to provide plants with Fe nutrition to enhance their growth under low-iron conditions. At the same time, the siderophores produced by PGPR bind the iron in order to reduce the Fe availability of fungal pathogens and efficiently prevent propagation [42]. In this study, the bacteria that produced the most siderophores were R25 and R325 (Figure 1c, Table 4). Many studies have shown that siderophore-producing bacteria can help plants to alleviate heavy metal stress and reduce the accumulation of heavy metals in plants [43]. The potential of the siderophore-producing bacteria in this study to aid in stress resistance needs to be further investigated. Compared with the application of a single bacterium, the combination formed by multiple bacteria with different functions is more stable. In comparison to diverse synthetic communities, lower-diversity communities are more likely to establish phenotype–genotype relationships and demonstrate higher reproducibility [44]. Therefore, we combined the above six bacteria and compared the growth-promoting effects of different combinations and single bacteria through several rounds of potting experiments (Tables S2–S4), eliminated the bacteria that had negative effects in the combinations, and finally obtained the three combinations with the best growth-promoting effects. The traditional screening and characterization method used in this study is less expensive than the popular method of establishing SynComs through histology. Compared with the results predicted via mathematical modeling, the combinations of bacteria in this study were all obtained via repeated practice, and the repeated combinations and splits among the six bacteria also ensured the stability of the results obtained.

### 4.2. The Bacterial Combinations Can Enhance the Photosynthetic Capacity of Tomato Seedlings

Promoting photosynthesis is a crucial element in achieving high crop yields in fruit and vegetable production [45]. *RbcL*, *RbcS* and *RCA* are responsible for controlling the synthesis of the Rubisco enzyme [46,47], while the Calvin cycle depends on the involvement of *GAPDH*, *SBPase* and *FBPase* [48,49,50]. *FBA* regulates the glycolytic pathways [51]. Simultaneous inoculation with bushy mycorrhizal fungi and inter-root bacteria has been proven to enhance the efficiency of photosynthesis and increase the content of photosynthetic pigments, leading to the growth promotion of tobacco plants [52]. Under conditions of drought stress, PGPR can modify the fundamental structure and functional characteristics of a plant’s photosynthetic system by generating phytohormones that enhance stomatal conductance, the photosynthetic rate, and reduce the plant’s transpiration rate [53]. In this study, there was no significant association between the net photosynthetic rate and intercellular carbon dioxide concentration (Figure 3), which may have been influenced by the mesophyll conductance. Therefore, to confirm the enhancement of photosynthesis by growth-promoting bacteria, we analyzed the relative expression of the mentioned genes that are linked with photosynthetic carbon assimilation based on the observed photosynthetic parameters (Figure 4). The expression of genes responsible for the formation of the Rubisco enzyme, Calvin cycle, and glycolysis increased in all treatment groups, significantly. In particular, the expression of all carbon assimilation genes was considerably high amongst the T5 treatment group, indicating that the two bacteria, R62 and R325, possibly played a crucial role. This study offers evidence, at the molecular level, that the combination of bacteria boosts photosynthetic rates and stimulates plant growth.

### 4.3. The Bacterial Combinations Potentially Improve the Resistance of Tomato Plants to Stresses

During the process of evolution, plants have developed an endogenous antioxidant defense system to reduce the oxidative damage caused by environmental stresses. Polyphenol oxidase (PPO) and peroxidase (POD) are crucial components of the enzymatic antioxidant defense system. The aim of this study was to explore the potential ability of bacterial combinations to induce resistance. In the present study, a slight decrease in POD enzyme activity was observed in the T8 group compared to the control group (Figure 5). Among them, both the T5 and T8 groups contained the siderophore-producing bacterium R325, which might have been caused by the competition between the bacteria R317 and R325 in the T8 treatment for the utilization of iron carriers, which needs to be further explored. Previous research indicates that *Pseudomonas stutzeri* and *Pseudomonas putida* increase the activities of polyphenol oxidase, phenylalanine deaminase, superoxide dismutase, peroxidase, and catalase in soybean infected with charcoal rot disease, as well as enhance the utilization of nutrients in the inter-root zone of infected soils [54]; this is also in agreement with the results of the present study, suggesting that the combination of bacteria in the present experiment may have the potential to improve tomato resistance to soil-borne diseases.

### 4.4. The Bacterial Combinations Soaking Seeds Can Increase Seed Vigor

Seed pre-treatment is an effective and time-saving method of signaling the plant at an early stage to promote growth and enhance resistance. This technique has been extensively utilized in major crops, including rice and tomato [55]. Previous studies demonstrated that the combination of *Azotobacter*, PSB, and *Pseudomonas* aeruginosa significantly enhanced growth parameters such as the total biomass and total chlorophyll of Coriander (*Coriandrum sativum* L.) compared to the control. [56]. Soaking maize seeds in a solution of three biotrophic bacteria (*P. fluorescens*, *P. putida*, and *A. lipoferum*) and chitosan resulted in improved germination [57]. The present study found similar results to previous studies, which demonstrated that soaking seeds with PGPR enhanced their vigor (Figure 6). In this soaking test, the seeds used were from the same batch and the T8 treatment group showed the highest germination percentage in the first seven days, while the germination potential was relatively low on the 10th day; this could be attributed to the progressive effect of the metabolites of the two bacteria on the seeds in the T8 treatment group.

This experiment primarily confirmed the impact of bacteria combinations on the growth of tomato seedlings in pots, but the effects of the three bacteria combinations on tomato plant growth need to be validated under real production conditions. The four bacteria (R62, R219, R317, and R325) in combination were non-antagonistic. Further experiments should adjust the quantity and concentration of these bacteria to confirm their potential to promote growth in tomato plants during their flowering and fruiting stage. The objective of this is to identify the most effective bacterial fertilizer composition for field use. Assessing changes in the soil enzyme activity and soil microbial community structure is imperative following the field application of combined bacteria. Furthermore, while R25 and R41 did not exhibit efficacy when combined with other bacteria, they demonstrated the capacity to promote growth when utilized individually; this was particularly the case for R25, which requires further exploration.

## Figures and Tables

**Table 1:** Isolates screened using LB medium.

Number	Nomenclature	Number	Nomenclature
R17	*Agrobacterium radiobacter*	F3-3-1	*Bacillus altitudinis*
R111	*Pseudochrobactrum asaccharolyticum*	R335	*Bacillus aryabhattai*
R328	*Pseudomonas mediterranea*	R33A	*Bacillus cereus*
R73	*Pseudomonas asiatica*	R43	*Bacillus flexus*
R27	*Pseudomonas composti*	R65A	*Bacillus halotolerans*
R219	*Pseudomonas corrugata*	R25	*Bacillus tequilensis*
R322A	*Pseudomonas mendocina*	R71B1	*Bacillus wiedmannii*
R74	*Pseudomonas stutzeri*	R32	*Paenibacillus dongdonensis*
R77	*Pseudomonas zhaodongensis*	R713	*Microbacterium aerolatum*
R317	*Advenella kashmirensis* subsp. *methylica*	R210	*Microbacterium esteraromaticum*
R511	*Alcaligenes faecalis* subsp. *phenolicus*	R24	*Microbacterium oxydans*
R75	*Bosea thiooxidans*	R72A	*Microbacterium shaanxiense*
F1-2	*Brucella cytisi*	R41	*Arthrobacter pokkalii*
R54	*Proteus mirabilis*	R37	*Brevibacterium sediminis*
R62	*Providencia vermicola*	R115	*Leucobacter aridicollis*
R217	*Sphingobacterium mizutaii*	R110	*Leucobacter iarius*
R325	*Empedobacter brevis*	F4-1	*Rhodococcus biphenylivorans*
R112A	*Myroides odoratimimus* subsp. *xuanwuensis*	R336	*Rhodococcus sovatensis*

**Table 2:** Capacity of different isolates to produce IAA.

Number	OD_600_	IAA Production (μg·mL^−1^)	Number	OD_600_	IAA Production (μg·mL^−1^)
F1-2	0.061	1.60	R75	0.060	1.54
F2-3	0.071	2.20	R77	0.088	3.22
F3-3-1	0.079	2.69	R79	0.070	2.14
F4-1	0.065	1.84	R110	0.077	2.57
F8-5	0.069	2.08	R111	0.069	2.08
R17	0.067	1.96	R112A	0.071	2.20
R23	0.083	2.93	R112B	0.079	2.69
R24	0.068	2.02	R115	0.069	2.08
R25	0.108	4.43	R118	0.078	2.63
R27	0.098	3.83	R210	0.080	2.75
R32	0.075	2.45	R217	0.065	1.84
R33A	0.086	3.11	R219	0.090	3.35
R37	0.089	3.29	R312	0.061	1.60
R41	0.070	2.14	R312B	0.075	2.45
R42	0.065	1.84	R317	0.199	9.92
R43	0.067	1.96	R320	0.072	2.27
R54	0.100	3.95	R322A	0.076	2.51
R62	0.159	7.51	R325	0.067	1.96
R65A	0.073	2.33	R328	0.080	2.75
R71B1	0.066	1.90	R335	0.070	2.14
R71B2	0.084	2.99	R336	0.070	2.14
R72A	0.072	2.27	R510	0.066	1.90
R73	0.080	2.75	R511	0.068	2.02
R74	0.070	2.14	R713	0.067	1.96

**Table 3:** Phosphorus solubility of different isolates.

Number	D (cm)	d (cm)	D/d	Number	D (cm)	d (cm)	D/d
R118	1.20	0.50	2.40	R77	0.90	0.65	1.38
R17	0.85	0.35	2.43	R65A	1.20	0.55	2.18
R111	0.80	0.35	2.29	R511	0.70	0.55	1.27
R24	1.30	0.65	2.00	R41	0.90	0.30	3.00
R23	0.70	0.45	1.56	R43	1.00	0.50	2.00
R25	0.90	0.40	2.25	R62	0.60	0.40	1.50
R210	1.30	0.85	1.53	R322A	1.30	1.10	1.18
F1-2	0.90	0.60	1.50	R219	1.00	0.30	3.33
F3-3-1	1.60	0.95	1.68	R328	1.10	0.55	2.00
R32	1.50	0.85	1.77	R335	1.20	0.65	1.85
R317	0.40	0.15	2.67	R33A	1.00	0.50	2.00

D: Diameter of the dissolved phosphorus ring; d: Diameter of the strain.

**Table 4:** Capacity of different isolates to produce siderophores.

Number	OD_680_	Number	OD_680_
R325	3.1275	R112B	2.1290
R25	3.0795	R322A	2.1045
F4-1	3.0620	R75	2.0435
R62	3.0220	R511	2.0410
R336	3.0205	R54	2.0245
R17	3.0010	R219	1.8840
R317	2.8160	R71B1	1.8315
R110	2.7540	F3-3-1	1.6190
R27	2.2120	R713	1.5740
R65A	2.1370	R77	1.4970

## Data Availability

All the data of this study have been included in this article.

## Supplementary Materials

The following supporting information can be downloaded at: https://www.mdpi.com/article/10.3390/plants12234000/s1, Table S1: Primers for qRT-PCR; Table S2: Effects of the growth-promoting bacteria combination on the biomass and growth of tomato seedlings (The first trial); Table S3: Effects of the growth-promoting bacteria combination on the biomass and growth of tomato seedlings (The second trial); Table S4: The quality of tomato seedlings treated by the third batch of growth-promoting bacteria combination(The third trial).
