# Supplementary material for: Assembly of Tomato Rhizobacteria from Different Functional Groups Improves Seedling Photosynthesis and Growth
Source: Plants (Basel). 2023 Nov 28;12(23):4000. doi: 10.3390/plants12234000 (PMC10708133; doi:10.3390/plants12234000)
Supplement: Supplementary file 1 [file plants-12-04000-s001.zip › plants-2728696-SI.pdf]

Table S1 Primers for qRT-PCR

Gene name	Primer sequence（5'-3'）
RbcL	F: GCGAATTCTGGTCAGGTTGA
	R: ACCTCT GTGTTCCCTTGAT
RbcS	F: CGTGAGAACAACAAGTCACC
	R: TCAGTGCACCCAAACATAGG
RCA	F: AGCCAAGGTCTTCGCCAATA
	R: TGGGCAACGTTAAGAAGTTC
GAPDH	F: TGTTTAAGTATGATAGTGTACACGG
	R: AACAGCCTTCTCACCAAAGA
FBPase	F: AGACTCCATTTTTATTTGCCTTGG
	R: CCTCCATTAAATGACCTTCTCTTT
SBPase	F: GATTACGCCAAGCTGATCGA
	R: TTTACATCAGGCACCATTCC
FDA	F: TTTGGACTCCTCAAAGTACCCTTGG
	R: CCGGTATTCCCAACGACAAG
EF-1a	F: ATTGGAAATGGATATGCTCCA
	R: TCCTTACCTGAACGCCTGTCA

Table S2 Effects of the growth-promoting bacteria combination on the biomass and growth of tomato seedlings (The first trial)

Combination	Fresh weight	Dry weight	SPAD	Plant height	Stem diameter	Seedling index
CK	0.662±0.0821c	0.087±0.0096c	39.660±4.7917	6.580±0.3680bc	1.174±0.0741c	0.031±0.0034d
MIX-R25	1.733±0.0790a	0.366±0.0148a	40.740±0.4833	8.500±0.3701a	2.956±0.0835a	0.219±0.0106a
MIX-R41	1.569±0.1089a	0.320±0.0214a	37.160±1.5510	8.620±0.2557a	2.912±0.0397a	0.179±0.0136b
MIX-R62	0.655±0.0537c	0.068±0.0044c	41.060±2.1913	6.640±0.2294bc	1.188±0.0663c	0.023±0.0013d
MIX-R219	1.104±0.0940b	0.205±0.0143b	44.060±2.7180	7.440±0.2581bc	1.480±0.0518b	0.077±0.0054c
MIX-R317	0.916±0.1487bc	0.118±0.0049c	37.000±2.6783	7.600±0.0949ab	1.176±0.0532c	0.038±0.0010d
MIX-R325	1.030±0.1353b	0.204±0.0273b	43.920±1.2706	7.040±0.5555bc	1.492±0.0604b	0.081±0.0121c
MIX	0.619±0.1530c	0.081±0.0085c	43.040±2.6322	6.420±0.4067c	1.148±0.0392c	0.028±0.0035d

Note: The data in the table are mean ± standard error (n=12), and different letters indicate significant differences ($P < 0.05$).

Table S3 Effects of the growth-promoting bacteria combination on the biomass and growth of tomato seedlings (The second trial)

Combination	Fresh weight	Dry weight	SPAD	Plant height	Stem diameter	Seedling index
CK	1.065±0.1289bc	0.070±0.0068d	34.400±1.4958d	8.817±0.1558a	1.163±0.1072d	0.015±0.0026d
R62+R219+R317+R325	1.665±0.1191a	0.220±0.0229a	49.600±1.4679a	7.900±0.6826b	3.457±0.1073a	0.125±0.0115a
R62+219+317	1.650±0.1218a	0.205±0.0175ab	48.033±1.6405b	7.433±0.6042b	3.117±0.1483b	0.114±0.0109a
R62+219+325	1.543±0.0943ab	0.179±0.0228b	46.133±2.664b	8.183±0.2522b	3.077±0.1410c	0.086±0.0143ab
R62+317+325	1.322±0.0811b	0.122±0.0090c	45.183±1.2682b	6.933±0.5760c	2.947±0.0701c	0.067±0.0047bc
R219+317+325	1.276±0.1067b	0.138±0.0109bc	45.800±1.7812b	7.383±0.2798c	2.973±0.1636c	0.072±0.0090bc
MIX-R41	1.340±0.1054b	0.130±0.0082bc	43.100±1.9115c	8.017±0.4370b	2.870±0.0471c	0.065±0.0041bc
MIX-R25	1.398±0.0809b	0.124±0.0053c	47.150±1.5880b	8.583±0.4549a	2.883±0.0847c	0.061±0.0044c
R325	1.530±0.0786ab	0.148±0.0038bc	47.817±1.5690b	7.000±0.3642c	3.090±0.0914c	0.083±0.0026b
R317	1.419±0.1267b	0.132±0.0055bc	45.633±0.9186b	7.400±0.3958b	3.115±0.1220b	0.074±0.0036b
R219	1.419±0.1141b	0.124±0.0058c	45.800±0.9306b	6.917±0.5192c	2.823±0.1736c	0.067±0.0051bc
R62	1.477±0.0604ab	0.125±0.0050c	45.367±1.1543b	7.483±0.4686b	2.977±0.0996c	0.063±0.0024bc
R41	0.892±0.16890c	0.098±0.0109cd	45.000±1.7624b	7.617±0.5406b	2.773±0.1069c	0.050±0.0074cd
R25	1.572±0.0853ab	0.163±0.0094b	46.250±1.3904b	7.683±0.2810b	3.062±0.1343c	0.081±0.0071b

Note: The data in the table are mean ± standard error (n=12), and different letters indicate significant differences ($P < 0.05$).

Table S4 The quality of tomato seedlings treated by the third batch of growth-promoting bacteria combination(The third trial)

Combination	Fresh weight	Dry weight	SPAD	Plant height	Stem diameter	Seedling index
CK	1.786±0.1321d	0.193±0.0029d	36.933±1.0291b	7.633±0.1498c	2.650±0.0832b	0.103±0.0019cd
R62+R219+R317+R325(T1)	4.304±0.1440a	0.458±0.0017ab	46.367±1.0541a	9.583±0.3420ab	3.658±0.0611a	0.281±0.0029ab
R62+R219+R317(T2)	3.540±0.1248cd	0.409±0.0011bc	46.400±1.0541a	9.533±0.3528ab	3.505±0.0904a	0.242±0.0012bc
R62+R219(T3)	3.612±0.0970c	0.385±0.0024cd	46.578±1.3038a	7.833±0.4602bc	3.525±0.1335a	0.258±0.0018b
R62+R317(T4)	4.214±0.1865ab	0.455±0.0028ab	46.117±1.1511a	9.967±0.3922a	3.762±0.0741a	0.262±0.0027b
R62+R325(T5)	3.976±0.1285b	0.487±0.0033a	45.867±0.5631a	9.233±0.5835b	3.583±0.0870a	0.312±0.0059a
R219+R317(T6)	3.826±0.1580bc	0.420±0.0030b	46.067±0.6270a	9.333±0.2246b	3.583±0.0970a	0.225±0.0032c
R219+R325(T7)	3.590±0.0828c	0.393±0.0029c	47.833±0.2552a	8.400±0.2608b	3.590±0.0879a	0.227±0.0032c
R317+R325(T8)	3.646±0.1200c	0.455±0.0028ab	46.850±0.6913a	8.850±0.5012b	3.468±0.0865a	0.276±0.0042ab

Note: The data in the table are mean ± standard error (n=12), and different letters indicate significant differences ($P < 0.05$).
